# Peripheral Protein Quality Control as a Novel Drug Target for CFTR Stabilizer

**DOI:** 10.3389/fphar.2018.01100

**Published:** 2018-09-27

**Authors:** Ryosuke Fukuda, Tsukasa Okiyoneda

**Affiliations:** Department of Biomedical Chemistry, School of Science and Technology, Kwansei Gakuin University, Nishinomiya, Japan

**Keywords:** peripheral QC, CFTR, stabilizer, ubiquitination, RFFL

## Abstract

Conformationally defective cystic fibrosis transmembrane conductance regulator (CFTR) including rescued ΔF508-CFTR is rapidly eliminated from the plasma membrane (PM) even in the presence of a CFTR corrector and potentiator, limiting the therapeutic effort of the combination therapy. CFTR elimination from the PM is determined by the conformation-dependent ubiquitination as a part of the peripheral quality control (PQC) mechanism. Recently, the molecular machineries responsible for the CFTR PQC mechanism which includes molecular chaperones and ubiquitination enzymes have been revealed. This review summarizes the molecular mechanism of the CFTR PQC and discusses the possibility that the peripheral ubiquitination mechanism becomes a novel drug target to develop the CFTR stabilizer as a novel class of CFTR modulator.

## Introduction

**FIGURE 1:**
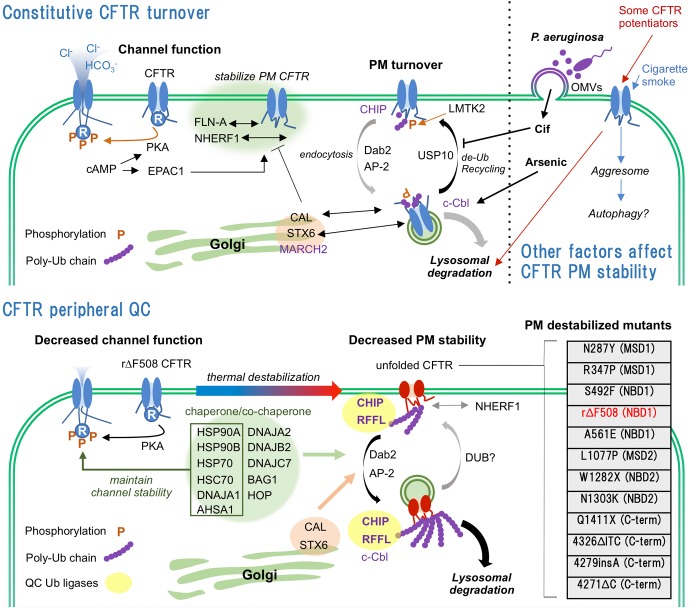
Constitutive turnover and PQC of CFTR. **(Upper)** WT-CFTR is stabilized at the PM by interaction with FLN-A and NHERF1. Internalization of WT-CFTR is regulated by LMTK2-mediated phosphorylation and CHIP-mediated ubiquitination. Predominant endocytosed WT-CFTR is recycled back to the PM by USP10 mediated de-ubiquitination. Golgi localized CAL complex promotes lysosomal sorting of CFTR. CFTR PM stability is deteriorated by infection, CS and CFTR potentiators. **(Lower)** Unfolded rΔF508-CFTR at the PM is ubiquitinated by PQC Ub ligases CHIP and RFFL, and rapidly internalized probably by CME mediated by DAB2 and AP-2. Chaperone/co-chaperone complex also facilitates the unfolded CFTR internalization while it helps the channel functionality at the PM. Internalized unfolded CFTR could be ubiquitinated at endosomes and targeted to lysosomal degradation. CAL also facilitates the lysosomal degradation of internalized CFTR. PM destabilizing CFTR mutations and their locations are listed (Veit et al., 2016a, Cystic Fibrosis Genetic Consortium Database). N287Y mutation increases CFTR endocytosis without affecting maturation (Silvis et al., 2003). R347P, S492F, rΔF508, A561E, L1077P, and N1303K mutations induce severe maturation defect and PM instability (Van Goor et al., 2014). N287Y and L1077P are localized at intracellular loop 2 (ICL2) of MSD1 and ICL4 of MSD2, respectively. Both mutations are predicted to destabilize the MSD1-NBD2 and MSD2-NBD1 interactions that define CFTR conformational stability.

Cystic fibrosis (CF) is one of the most lethal autosomal-recessive diseases caused by mutation in CFTR (Lopes-Pacheco, 2016). CFTR mutations are classified as I–VII according to their properties (I–protein synthesis defect, II-maturation defect, III-gating defect, IV-conductance defect, V-reduced quantity, VI-reduced PM stability, VII-no mRNA transcription). The most prevalent CF causing mutation, ΔF508, was classically categorized as class II mutation. However, rescued ΔF508 (rΔF508)-CFTR by corrector (e.g., VX-809/lumacaftor) or low temperature culture shows class III and VI phenotypes (Dalemans et al., 1991; Veit et al., 2016a). Although drug targets of the class II or III mutations are well studied, that of the class VI mutation are not because the mechanism of CFTR PM stability regulation are still veiled by numerous undefined molecules involved in CFTR PQC system. In this review, we summarize accumulated findings regarding the CFTR PQC from the molecular and environmental aspects and also discuss the potential of recently identified PQC machineries including endocytic adaptors and ubiquitination enzymes as targets for CFTR stabilizer which anchors the functional channel at the PM and reduces the degradation (**Figure 1**).

## CFTR Instability at the PM

Nascent wild-type (WT) CFTR is *N*-glycosylated at the endoplasmic reticulum (ER) during translation and folded by the aid of chaperones such as calnexin (CNX), HSP70 and HSP90 (Amaral, 2004; Kleizen et al., 2005; Okiyoneda et al., 2008; Rosser et al., 2008; Glozman et al., 2009; Kim and Skach, 2012). Properly folded CFTR is then sorted to the Golgi apparatus and processed to complex glycosylation while misfolded CFTR is retained in the ER and consequently degraded by ER-associated degradation (ERAD). The CFTR ERAD is associated with several ER QC processes such as chaperones binding, ubiquitination and retro-translocation from the ER to cytosol (Younger et al., 2006; Turnbull et al., 2007; Morito et al., 2008; Grove et al., 2011; Nery et al., 2011; El Khouri et al., 2013; Matsumura et al., 2013; Ernst et al., 2016; Gong et al., 2016; McClure et al., 2016; Ramachandran et al., 2016; Hou et al., 2018). Properly folded and matured CFTR is trafficked to the PM to function as an ATP-regulated ion-channel (Chen et al., 2000).

The CFTR is internalized by clathrin-mediated endocytosis (CME) and recycled back to the PM. Conformationally defective CFTR produced by genetic mutations (e.g., ΔF508, T70) and/or environmental stresses (e.g., heat) selectively undergoes ubiquitination at the PM by PQC machineries. The ubiquitinated CFTR is rapidly internalized and delivered to lysosome for degradation (Sharma et al., 2004; Okiyoneda et al., 2010). Internalized CFTR could be de-ubiquitinated at endosomes by deubiquitinase (DUB) and recycled back to the PM depending on the conformational states. The class VI mutations render the CFTR unstable at the PM. Additionally, the class I, II and some class III CFTR mutants also show PM instability (Lukacs et al., 1993; Haardt et al., 1999; Silvis et al., 2003; Wang et al., 2014; Veit et al., 2016a). *N*-glycosylation, especially the core-glycosylation, determines the CFTR PM stability likely by affecting the CFTR conformational stability (Glozman et al., 2009; Cholon et al., 2010). Protein translation kinetics is also a significant factor that modulates proper co-translational folding. Knock down (KD) of ribosomal protein L12 (RPL12) increases ΔF508-CFTR PM expression and stability (Veit et al., 2016b). RPL12 KD might affect protein translation kinetics associated with co-translational protein folding efficiency (Buhr et al., 2016) and thereby improve CFTR thermodynamic stability which also determines the CFTR PM stability (Okiyoneda et al., 2010; Rabeh et al., 2012). Thus, correcting the CFTR structural defects at the ER could improve the PM stability.

## Environmental Stresses Affecting the CFTR PM Stability

### Infection and Inflammation

The CFTR loss of function induces airway surface liquid (ASL) dysregulation which impairs clearance of infected bacteria and/or fungi, and increases the concentration of other soluble signal mediators such as cytokines, chemokines and growth factors. *Pseudomonas aeruginosa* (PA) is one of the most common bacteria found in CF respiratory tissue and responsible for lung injury in CF (Koch, 2002; Bhagirath et al., 2016). PA destabilizes PM CFTR by inhibiting endocytic recycling (Swiatecka-Urban et al., 2006). PA secretes CFTR inhibitory factor (Cif) that stabilizes complex formation of ubiquitin (Ub) specific peptidase 10 (USP10) and GTPase activating protein (SH3 domain) binding protein 1 (G3BP1) and inhibits CFTR-USP10 interaction. Cif inhibits internalized CFTR sorting to recycling pathway by suppressing USP10 dependent CFTR de-ubiquitination at endosome, resulting in the lysosomal degradation of WT-CFTR (Bomberger et al., 2011). PA also activates transforming growth factor β1 (TGF-β1) signaling that is an important modifier of lung disease severity in CF (Harris et al., 2011). TGF-β1 inhibits functional PM expression of WT-CFTR and ΔF508-CFTR by reducing mRNA level (Snodgrass et al., 2013; Sun et al., 2014) although its role in the PQC remains unknown.

### Heavy Metals

More than 10 ppb of arsenic induces the WT-CFTR ubiquitination and lysosomal degradation via c-Cbl in CF bronchial epithelial (CFBE) cells (Bomberger et al., 2012). Importantly, the phenotype of arsenic toxicity overlaps with CF patient (Bomberger et al., 2012; Mazumdar et al., 2015). Cadmium (Cd) is a major component of cigarette smoke (CS), and its inhalation is associated with decreased pulmonary function and chronic obstructive pulmonary disease. Cd reduces CFTR PM level, but it remains unknown if it reduces the PM stability (Rennolds et al., 2010).

### Cigarette Smoke

Cigarette smoke is a major risk factor of chronic obstructive pulmonary disease and interferes with CFTR functionality. Ten minutes of CS exposure transiently suppresses CFTR function, induces internalization and decreases ASL height in human bronchial epithelial (HBE) cells (Clunes et al., 2012). CS promotes CFTR internalization in BHK cells and results in increased insolubility of CFTR and colocalization with vimentin, a filament protein associated with aggresome Ca^2+^ dependently. This observation suggesting that CS induces PM CFTR destabilization by stimulating internalization and aggregation in addition to suppressing CFTR functionality (Clunes et al., 2012; Rasmussen et al., 2014).

## Molecular Machineries Determining the CFTR PM Stability

### Endocytosis Adaptors and Tethering Factors

Endocytosis is the critical step of elimination of PM CFTR as a part of PQC and is regulated by several molecules. WT-CFTR is internalized slowly by CME while misfolded rΔF508-CFTR endocytosis is accelerated (Sharma et al., 2004; Swiatecka-Urban et al., 2005; Varga et al., 2008; Okiyoneda et al., 2010). KD of CME adaptor AP-2 μ2 subunit or disabled 2 (DAB2) stabilizes rΔF508-CFTR at the PM by inhibiting endocytosis (Fu et al., 2012, 2015).

CFTR has a postsynaptic density 95, disks large, zonula occludens-1 (PDZ) binding motif at C-terminus and binds with Na^+^/H^+^ exchanger regulatory factor (NHERF1) PDZ domain. NHERF1 tethers CFTR with Ezrin and works as a scaffold protein that supports CFTR efficient channel activation and apical PM localization (Favia et al., 2010; Arora et al., 2014; Loureiro et al., 2015). NHERF1 also binds to misfolded ΔF508-CFTR and increases the PM stability by inhibiting carboxy terminus of HSP70-interacting protein (CHIP) Ub ligase interaction (Loureiro et al., 2015). An exchange protein directly activated by cAMP1 (EPAC1) selective activating cAMP analog 007-AM promotes WT-CFTR and NHERF1 interaction and increases CFTR PM stability in CFBE cells by suppressing endocytosis (Lobo et al., 2016). EPAC1 activation can rescue ΔF508-CFTR PM expression, and its effect is further improved with VX-809 combination (Lobo et al., 2016).

The CFTR-associated ligand (CAL) negatively regulates ΔF508-CFTR PM abundance through its PDZ domain (Wolde et al., 2007). CAL inhibition enhances the functional stability of ΔF508-CFTR at the apical PM, implying an attractive therapeutic target for CFTR PM stabilizer (Cushing et al., 2010). However, CAL also interacts with syntaxin 6 (STX6) and Golgi-localized E3-ligase membrane associated RING-CH type finger 2 (MARCH2) and regulates WT-CFTR PM expression (Wolde et al., 2007; Cheng and Guggino, 2013).

Filamin-A (FLN-A) is a membrane tethered actin adaptor protein and interacts with CFTR N-terminus region. S13F mutation of CFTR compromises FLN-A binding and consequently destabilizes the PM CFTR (Thelin et al., 2007). FLN-A binds with both WT and rΔF508-CFTR at similar level, however, its contribution to the CFTR PQC remains unclear.

### Protein Kinases

The CFTR PM stability is regulated by phosphorylation. CFTR is predominantly phosphorylated at the R domain and also at nucleotide binding domain 1 (NBD1) and C-terminus residues by protein kinase A (PKA), protein kinase C (PKC), casein kinase II (CK2) and AMP-activated protein kinase (AMPK) for the channel function (Chappe et al., 2003; Kongsuphol et al., 2009; Luz et al., 2011). CK2 is predicted to regulate CFTR PM stability by phosphorylation at Thr-1471 where NHERF1 could interact (Venerando et al., 2013). Lemur tyrosine kinase 2 (LMTK2) phosphorylates CFTR at Ser-737 (Wang and Brautigan, 2006) and its KD or mutation at CFTR Ser-737 suppresses the endocytosis and increases CFTR PM density and stability (Luz et al., 2014). However, LMKT2 KD only modestly improves the PM function of rΔF508-CFTR (Luz et al., 2014). Spleen tyrosine kinase (SYK) phosphorylates CFTR at Tyr-512 and decreases CFTR PM levels possibly by triggering endocytosis (Luz et al., 2011; Mendes et al., 2011). Mixed-lineage kinase 3 (MLK3) pathway regulates not only ΔF508-CFTR ERQC, but also the PQC by regulating the CFTR proteostasis (Hegde et al., 2015). Inhibition of MLK3 pathway could regulate ΔF508-CFTR folding/degradation switch by impairing interaction with PQC machinery such as HSP70/HSP90 Organizing Protein (HOP) (Hegde et al., 2015).

### Chaperones

Molecular chaperones selectively interact with and stabilize unfolded or partially folded protein to acquire a functionally active conformation. Nascent CFTR interacts with a panel of chaperones and co-chaperones including HSC70, HSP70, HSP90, and CNX at the ER (Yang et al., 1993; Pind et al., 1994; Loo et al., 1998; Meacham et al., 1999; Okiyoneda et al., 2004). Even at the post-ER compartments, conformationally defective CFTR such as unfolded rΔF508-CFTR is recognized by chaperone/co-chaperone complex (Okiyoneda et al., 2010). HSC70/HSP90 complex selectively interacts with unfolded rΔF508-CFTR at the post-Golgi and this interaction is crucial for the unfolding dependent ubiquitination (Okiyoneda et al., 2010). KD of HSC70/HSP90 complex (HSP90, HSC70, HOP, AHA1, DNAJB2, DNAJA1, BAG1) increases the rΔF508-CFTR PM stability in HeLa cells (Okiyoneda et al., 2010). The HSC70/HSP90 complex is also essential for maintaining kinetic and thermodynamic stability of rΔF508-CFTR at the PM by reshaping the CFTR conformation during energetic destabilization (Bagdany et al., 2017). This chaperone activity also maintains rΔF508-CFTR channel function at the PM (Bagdany et al., 2017). Thus, modulating the chaperone activity would be a viable target for attenuating the ubiquitination and for stabilizing the CFTR function at the PM.

### Ubiquitination Enzymes

Ubiquitination determines CFTR elimination not only at the ER, but also from the PM. Ubiquitination is mediated by a sequential action of E1, E2, and E3 enzymes and this modification could be removed by DUB. Specifically, E3 Ub ligase has been proposed to determine the substrate specificity. CHIP is the first identified E3 ligase responsible for the CFTR PQC (Okiyoneda et al., 2010). Consistent with the action at the ER (Meacham et al., 2001), CHIP selectively interacts with unfolded ΔF508-CFTR at the post-Golgi through the HSC70/HSP90 chaperones. CHIP KD reduces the ubiquitination of unfolded ΔF508-CFTR, resulting in the decelerated endocytosis and lysosomal delivery in HeLa cells (Okiyoneda et al., 2010). CHIP KD also stabilizes rΔF508-CFTR at the PM of polarized CFBE cells (Fu et al., 2015).

E3 ligase c-Cbl may play a role in the CFTR peripheral QC, but its contribution could be modest since its KD slightly increases rΔF508-CFTR PM stability in CFBE cells (Cihil et al., 2013; Fu et al., 2015). c-Cbl also binds with WT-CFTR and decreases the PM stability without affecting the ubiquitination, suggesting that c-Cbl could regulate constitutive PM turnover of folded CFTR by inducing endocytosis through its C-terminus adaptor function (Ye et al., 2010).

Nedd4-2 is a member of homologous to the E6-AP carboxyl terminus (HECT) E3 which may regulate the CFTR PM expression. Nedd4-2 KD reduces ΔF508-CFTR ubiquitination at the ER, and increases the PM expression and function in CF pancreatic adenocarcinoma cell 1 (CFPAC1) and IB3-1 cells (Caohuy et al., 2009). Nedd4-2 binds both WT- and ΔF508-CFTR while its role in the WT-CFTR ubiquitination remains controversial (Koeppen et al., 2012). However, Nedd4-2 KD does not stabilize the PM rΔF508-CFTR in CFBE cells, implying its marginal contribution to the CFTR PQC (Koeppen et al., 2012; Fu et al., 2015). Nedd4-2 is unlikely a viable CF drug target because its knock out (KO) induces CF-like lung phenotype by excessive function of epithelial Na^+^ Channel (ENaC) (Kimura et al., 2011; Rotin and Staub, 2012).

A number of DUBs regulate the CFTR turnover. USP10, a DUB localized at early endosomes, interacts with WT-CFTR and reduces the CFTR poly-ubiquination in CFBE cells. The USP10-mediated deubiquitination enhances the endocytic recycling of WT-CFTR (Bomberger et al., 2009). The role of USP10 in the PM stability of conformationally defective CFTR such as rΔF508-CFTR remains unclear.

Recently, we have discovered RING finger and FYVE like domain containing E3 Ub protein ligase (RFFL) as a novel component of the CFTR PQC machineries by a comprehensive siRNA screen in CFBE cells (Okiyoneda et al., 2018). RFFL selectively recognizes unfolded rΔF508-CFTR through the disordered regions. RFFL promotes K63-linked poly-ubiquitination of the unfolded CFTR in post-Golgi, resulting in accelerated endocytosis and lysosomal degradation. Importantly, RFFL directly interacts with conformationally defective CFTR such as rΔF508-CFTR, but not with folded WT-CFTR at the PM and endosomes. Moreover, the RFFL-mediated ubiquitination is conformation dependent as it selectively ubiquitinates thermally unfolded NBD1. RFFL KD enhances the functional PM expression of rΔF508-CFTR in the presence of VX-809, and this effect is further improved by inhibiting the HSC70-dependent ubiquitination machinery. Thus, RFFL plays an important role in the chaperone-independent CFTR PQC mechanism in HBE cell models.

## CFTR Modulators Affecting the CFTR PM Stability

### Pharmacological Chaperones and Chemical Chaperones

Pharmacological chaperones affect the CFTR PM stability by direct stabilization. CFTR corrector VX-809 is the first food and drug administration (FDA) approved CFTR corrector in combination with VX-770/ivacaftor (known as Orkambi). VX-809 selectively improves the processing of misfolded CFTR by stabilizing NBD1-membrane spanning domain (MSD) interface but not other misfolded proteins such as human ether-à-go-go-related gene (hERG) mutants (Van Goor et al., 2011; Okiyoneda et al., 2013; Ren et al., 2013; Farinha et al., 2015). VX-809 repairs not only the CFTR folding defect at the ER but also the CFTR PM instability. VX-809 washout prolongs ΔF508-CFTR functional sustainability (Van Goor et al., 2011), suggesting that improvement of the CFTR folding at the ER could increase the thermal stability and proper co- and/or post-translational modifications that renders CFTR more energetic robust conformations even at the PM. VX-809 also promotes ΔF508-CFTR and NHERF1 interaction, that may increase the PM stability (Arora et al., 2014). C3 (CFcor-325/VRT-325) and C4 (Corr-4a) also extend rΔF508-CFTR PM stability in CFBE cells probably by directing binding (Wang et al., 2007; Varga et al., 2008) although their effect could be not specific to the conformationally defective CFTR (Van Goor et al., 2011). Chemical chaperones such as glycerol also increases the rΔF508-CFTR PM stability probably by non-specifically improving the conformational stability (Okiyoneda et al., 2013).

### CFTR Potentiators

The first FDA approved CFTR potentiator VX-770 improves the gating defect of some CFTR mutants. However, chronic VX-770 treatment destabilizes the PM rΔF508-CFTR in CFBE and ΔF508 homozygous CF patient HBE (CF-HBE) cells (Cholon et al., 2014; Veit et al., 2014). Importantly, chronic VX-770 treatment diminishes the VX-809 therapeutic efficacy by stimulating the elimination of PM rΔF508-CFTR (Cholon et al., 2014; Veit et al., 2014). In addition to VX-770, several CFTR potentiators including P1 (VRT-532) and P2 (PG-01) also decrease the rΔF508-CFTR PM stability (Veit et al., 2014). VX-770 and other potentiators could destabilize a variety of CFTR rare mutants referred to as CFTR2 mutants including E92K and L1077P at the PM (Avramescu et al., 2017). Thus, several CFTR potentiators may decrease the thermal stability of metastable mutant CFTR at the PM by inducing conformational change that positively affects for channel gating but negatively affects stability. High-throughput screening has identified several novel CFTR potentiators such as class A analog 4 (A04) and class P analog 12 (P12) that could not destabilize the PM rΔF508-CFTR (Phuan et al., 2015).

### Proteostasis Regulating Drugs

Proteostasis regulating drugs that affect array of proteins regulating CFTR folding and QC also affect the CFTR PM stability. Histone deacetylase (HDAC) inhibitor suberoylanilide hydroxamic acid (SAHA) alters expression of a subset of CF-interacting gene products (e.g., chaperones and DAB2) and sustains PM expression of ΔF508-CFTR in CFBE cells (Hutt et al., 2010). Tissue transglutaminase (TGM2) inhibitor cystamine also stabilizes ΔF508-CFTR at the PM of airway epithelial cells by restoring BECN1 interactome which is sequestrated by CFTR dysfunction (Luciani et al., 2012; Villella et al., 2013). MLK3 pathway inhibitor oxozeaenol has been reported to be effective in correcting the ΔF508-CFTR proteostasis defect in the primary HBE cells (Trzcinska-Daneluti et al., 2012). Oxozeaenol could stabilize ΔF508-CFTR at the PM as MLK3 KD reduces mature ΔF508-CFTR elimination by PQC (Hegde et al., 2015).

### Cavosonstat and CAL Inhibitor

HSP70/HSP90 Organizing Protein is adaptor protein which coordinates HSP70 and HSP90 function in protein folding and regulates CFTR maturation and PM stability (Odunuga et al., 2004; Okiyoneda et al., 2010). HOP *S*-nitrosylation by *S*-nitrosoglutathione (GSNO) induces HOP degradation and increases ΔF508-CFTR PM expression (Marozkina et al., 2010). Levels of *S*-nitrosothiols such as GSNO are low in CF airway (Grasemann et al., 1999) and *S*-nitrosothiol decreases the internalization rate of rΔF508-CFTR in HBE cells (Grasemann et al., 1999, 2000; Zaman et al., 2014). Cavosonstat (N91115) is an orally bioavailable inhibitor of GSNO reductase and restores GSNO levels (Donaldson et al., 2017). Cavosonstat is the first CFTR stabilizer in phase II trials, but it was not beneficial for improvement of lung function in combination with ivacaftor.

CFTR-associated ligand binds CFTR via a PDZ interaction domain and targets CFTR for lysosomal degradation (Cheng et al., 2004). CAL inhibition increases the PM stability of ΔF508-CFTR (Cushing et al., 2010) and cell penetrating CAL inhibiting peptide is established (Qian et al., 2015). CAL inhibitor has been developed as a cell surface CFTR stabilizer in pre-clinical level while its therapeutic efficacy and conformational selectivity remain unclear.

### Ub Ligase Inhibitors

RING finger protein 5 (RNF5/RMA1) is an ER associated E3 Ub ligase that regulates early stage CFTR proteostasis at the ER (Younger et al., 2006). A RNF5 inhibitor Inh-2 identified by homology modeling and virtual ligand screening causes significant rescue of ΔF508-CFTR in immortalized and primary HBE cells from CF patients (Sondo et al., 2018). Intriguingly, Inh-2 modestly increases mature ΔF508-CFTR half-life and this stabilization effect is further improved by VX-809. While the contribution of RNF5 in the CFTR peripheral QC remains unclear, RNF5 inhibitor may be useful to overcome the CFTR instability.

Currently, CHIP and RFFL are the only Ub ligases responsible for the CFTR peripheral QC (Okiyoneda et al., 2010, 2018). Thus, inhibiting their activity could selectively reduce the ubiquitination and elimination of unfolded CFTR from the PM, improving the limited efficacy of CF combination therapy. CHIP binds and regulates a number of substrates via chaperones (Connell et al., 2001). Moreover, inhibiting the CHIP activity induces deleterious effect as the CHIP KO mice result in the abnormal phenotypes including ataxia and pre-mature death^1^. In contrast, RFFL could bind and regulate a limited number of substrates because of its nature of direct binding to the CFTR through the disordered regions (Okiyoneda et al., 2018). More importantly, inhibiting the RFFL activity seems to have no venomousness since the RFFL KO mice exhibit no abnormal phenotype (Ahmed et al., 2009). Therefore, counteracting RFFL activity may provide a preferable therapeutic approach as a CFTR stabilizer that is a class of drugs that extends the PM resident time of CFTR class VI mutants. Although future studies are needed to validate the impact on ΔF508-CFTR in CF-HBE cells, developing agents selectively inhibiting RFFL-mediated CFTR ubiquitination may help improve the efficacy of CF pharmacological therapy.

## Conclusion and Perspective

Beside the progresses of CF pharmacological therapy, stabilizing the cell surface CFTR remains challenging and is necessary to improve the limited therapeutic efficacy. Recent studies have revealed some of the CFTR PQC mechanism eliminating the functional but conformationally defective CFTR from the PM. Understanding the CFTR PQC mechanism help the development of the CFTR stabilizer, a novel class of CFTR modulator necessary to establish the robust CF pharmacological therapy.

## Author Contributions

All authors listed have made a substantial, direct and intellectual contribution to the work, and approved it for publication.

## Conflict of Interest Statement

TO has a patent pending in Japan for methodology to identify inhibitors of RFFL-mediated CFTR ubiquitination (2017-047626). The remaining author declares that the research was conducted in the absence of any commercial or financial relationships that could be construed as a potential conflict of interest.

## References

[B1] Ahmed A. U. Moulin M. Coumailleau F. Wong W. W. Miasari M. Carter H. (2009). CARP2 deficiency does not alter induction of NF-kappaB by TNFalpha. *Curr. Biol.* 19 R15–R17. 10.1016/j.cub.2008.11.040 19138581

[B2] Amaral M. D. (2004). CFTR and chaperones: processing and degradation. *J. Mol. Neurosci.* 23 41–48. 10.1385/JMN:23:1-2:041 15126691

[B3] Arora K. Moon C. Zhang W. Yarlagadda S. Penmatsa H. Ren A. (2014). Stabilizing rescued surface-localized δf508 CFTR by potentiation of its interaction with Na( + )/H( + ) exchanger regulatory factor 1. *Biochemistry* 53 4169–4179. 10.1021/bi401263h 24945463 PMC4081048

[B4] Avramescu R. G. Kai Y. Xu H. Bidaud-Meynard A. Schnúr A. Frenkiel S. (2017). Mutation-specific downregulation of CFTR2 variants by gating potentiators. *Hum. Mol. Genet.* 26 4873–4885. 10.1093/hmg/ddx367 29040544 PMC5886047

[B5] Bagdany M. Veit G. Fukuda R. Avramescu R. G. Okiyoneda T. Baaklini I. (2017). Chaperones rescue the energetic landscape of mutant CFTR at single molecule and in cell. *Nat. Commun.* 8:398. 10.1038/s41467-017-00444-4 28855508 PMC5577305

[B6] Bhagirath A. Y. Li Y. Somayajula D. Dadashi M. Badr S. Duan K. (2016). Cystic fibrosis lung environment and *Pseudomonas aeruginosa* infection. *BMC Pulm. Med.* 16:174. 10.1186/s12890-016-0339-5 27919253 PMC5139081

[B7] Bomberger J. M. Barnaby R. L. Stanton B. A. (2009). The deubiquitinating enzyme USP10 regulates the post-endocytic sorting of cystic fibrosis transmembrane conductance regulator in airway epithelial cells. *J. Biol. Chem.* 284 18778–18789. 10.1074/jbc.M109.001685 19398555 PMC2707225

[B8] Bomberger J. M. Coutermarsh B. A. Barnaby R. L. Stanton B. A. (2012). Arsenic promotes ubiquitinylation and lysosomal degradation of cystic fibrosis transmembrane conductance regulator (CFTR) chloride channels in human airway epithelial cells. *J. Biol. Chem.* 287 17130–17139. 10.1074/jbc.M111.338855 22467879 PMC3366821

[B9] Bomberger J. M. Ye S. Maceachran D. P. Koeppen K. Barnaby R. L. O’Toole G. A. (2011). A *Pseudomonas aeruginosa* toxin that hijacks the host ubiquitin proteolytic system. *PLoS Pathog.* 7:e1001325. 10.1371/journal.ppat.1001325 21455491 PMC3063759

[B10] Buhr F. Jha S. Thommen M. Mittelstaet J. Kutz F. Schwalbe H. (2016). Synonymous codons direct cotranslational folding toward different protein conformations. *Mol. Cell.* 61 341–351. 10.1016/j.molcel.2016.01.008 26849192 PMC4745992

[B11] Caohuy H. Jozwik C. Pollard H. B. (2009). Rescue of DeltaF508-CFTR by the SGK1/Nedd4-2 signaling pathway. *J. Biol. Chem.* 284 25241–25253. 10.1074/jbc.M109.035345 19617352 PMC2757227

[B12] Chappe V. Hinkson D. A. Zhu T. Chang X. B. Riordan J. R. Hanrahan J. W. (2003). Phosphorylation of protein kinase C sites in NBD1 and the R domain control CFTR channel activation by PKA. *J. Physiol.* 548 39–52. 10.1113/jphysiol.2002.035790 12588899 PMC2342791

[B13] Chen E. Y. Bartlett M. C. Clarke D. M. (2000). Cystic fibrosis transmembrane conductance regulator has an altered structure when its maturation is inhibited. *Biochemistry* 39 3797–3803. 10.1021/bi992620m 10736180

[B14] Cheng J. Guggino W. (2013). Ubiquitination and degradation of CFTR by the E3 ubiquitin ligase MARCH2 through its association with adaptor proteins CAL and STX6. *PLoS One* 8:e68001. 10.1371/journal.pone.0068001 23818989 PMC3688601

[B15] Cheng J. Wang H. Guggino W. B. (2004). Modulation of mature cystic fibrosis transmembrane regulator protein by the PDZ domain protein CAL. *J. Biol. Chem.* 279 1892–1898. 10.1074/jbc.M308640200 14570915

[B16] Cholon D. M. O’Neal W. K. Randell S. H. Riordan J. R. Gentzsch M. (2010). Modulation of endocytic trafficking and apical stability of CFTR in primary human airway epithelial cultures. *Am. J. Physiol. Lung. Cell Mol. Physiol.* 298 L304–L314. 10.1152/ajplung.00016.2009 20008117 PMC2838667

[B17] Cholon D. M. Quinney N. L. Fulcher M. L. Esther C. R. Das J. Dokholyan N. V. (2014). Potentiator ivacaftor abrogates pharmacological correction of ΔF508 CFTR in cystic fibrosis. *Sci. Transl. Med.* 6:246ra96. 10.1126/scitranslmed.3008680 25101886 PMC4272825

[B18] Cihil K. M. Zimnik A. Swiatecka-Urban A. (2013). c-Cbl reduces stability of rescued ΔF508-CFTR in human airway epithelial cells: implications for cystic fibrosis treatment. *Commun. Integr. Biol.* 6:e23094. 10.4161/cib.23094 23750297 PMC3609839

[B19] Clunes L. A. Davies C. M. Coakley R. D. Aleksandrov A. A. Henderson A. G. Zeman K. L. (2012). Cigarette smoke exposure induces CFTR internalization and insolubility, leading to airway surface liquid dehydration. *FASEB J.* 26 533–545. 10.1096/fj.11-192377 21990373 PMC3290447

[B20] Connell P. Ballinger C. A. Jiang J. Wu Y. Thompson L. J. Höhfeld J. (2001). The co-chaperone CHIP regulates protein triage decisions mediated by heat-shock proteins. *Nat. Cell Biol.* 3 93–96. 10.1038/35050618 11146632

[B21] Cushing P. R. Vouilleme L. Pellegrini M. Boisguerin P. Madden D. R. (2010). A stabilizing influence: CAL PDZ inhibition extends the half-life of ΔF508-CFTR. *Angew. Chem. Int. Ed. Engl.* 49 9907–9911. 10.1002/anie.201005585 21105033 PMC3033200

[B22] Dalemans W. Barbry P. Champigny G. Jallat S. Dott K. Dreyer D. (1991). Altered chloride ion channel kinetics associated with the delta F508 cystic fibrosis mutation. *Nature* 354 526–528. 10.1038/354526a0 1722027

[B23] Donaldson S. H. Solomon G. M. Zeitlin P. L. Flume P. A. Casey A. McCoy K. (2017). Pharmacokinetics and safety of cavosonstat (N91115) in healthy and cystic fibrosis adults homozygous for *F*508DEL-CFTR. *J. Cyst. Fibros* 16 371–379. 10.1016/j.jcf.2017.01.009 28209466

[B24] El Khouri E. Le Pavec G. Toledano M. B. Delaunay-Moisan A. (2013). RNF185 is a novel E3 ligase of endoplasmic reticulum-associated degradation (ERAD) that targets cystic fibrosis transmembrane conductance regulator (CFTR). *J. Biol. Chem.* 288 31177–31191. 10.1074/jbc.M113.470500 24019521 PMC3829429

[B25] Ernst W. L. Shome K. Wu C. C. Gong X. Frizzell R. A. Aridor M. (2016). VAMP-associated Proteins (VAP) as receptors that couple cystic fibrosis transmembrane conductance regulator (CFTR). proteostasis with lipid homeostasis. *J. Biol. Chem.* 291 5206–5220. 10.1074/jbc.M115.692749 26740627 PMC4777854

[B26] Farinha C. M. Sousa M. Canato S. Schmidt A. Uliyakina I. Amaral M. D. (2015). Increased efficacy of VX-809 in different cellular systems results from an early stabilization effect of F508del-CFTR. *Pharmacol. Res. Perspect.* 3:e00152. 10.1002/prp2.152 26171232 PMC4492728

[B27] Favia M. Guerra L. Fanelli T. Cardone R. A. Monterisi S. Di Sole F. (2010). Na + /H + exchanger regulatory factor 1 overexpression-dependent increase of cytoskeleton organization is fundamental in the rescue of F508del cystic fibrosis transmembrane conductance regulator in human airway CFBE41o- cells. *Mol. Biol. Cell* 21 73–86. 10.1091/mbc.E09-03-0185 19889841 PMC2801722

[B28] Fu L. Rab A. Tang L. Bebok Z. Rowe S. M. Bartoszewski R. (2015). ΔF508 CFTR surface stability is regulated by DAB2 and CHIP-mediated ubiquitination in post-endocytic compartments. *PLoS One* 10:e0123131. 10.1371/journal.pone.0123131 25879443 PMC4399842

[B29] Fu L. Rab A. Tang L. P. Rowe S. M. Bebok Z. Collawn J. F. (2012). Dab2 is a key regulator of endocytosis and post-endocytic trafficking of the cystic fibrosis transmembrane conductance regulator. *Biochem. J.* 441 633–643. 10.1042/BJ20111566 21995445 PMC3646389

[B30] Glozman R. Okiyoneda T. Mulvihill C. M. Rini J. M. Barriere H. Lukacs G. L. (2009). N-glycans are direct determinants of CFTR folding and stability in secretory and endocytic membrane traffic. *J. Cell Biol.* 184 847–862. 10.1083/jcb.200808124 19307599 PMC2699153

[B31] Gong X. Ahner A. Roldan A. Lukacs G. L. Thibodeau P. H. Frizzell R. A. (2016). Non-native conformers of cystic fibrosis transmembrane conductance regulator NBD1 are recognized by Hsp27 and conjugated to SUMO-2 for degradation. *J. Biol. Chem.* 291 2004–2017. 10.1074/jbc.M115.685628 26627832 PMC4722474

[B32] Grasemann H. Gaston B. Fang K. Paul K. Ratjen F. (1999). Decreased levels of nitrosothiols in the lower airways of patients with cystic fibrosis and normal pulmonary function. *J. Pediatr.* 135 770–772. 10.1016/S0022-3476(99)70101-0 10586185

[B33] Grasemann H. Knauer N. Büscher R. Hübner K. Drazen J. M. Ratjen F. (2000). Airway nitric oxide levels in cystic fibrosis patients are related to a polymorphism in the neuronal nitric oxide synthase gene. *Am. J. Respir. Crit. Care Med.* 162 2172–2176. 10.1164/ajrccm.162.6.2003106 11112133

[B34] Grove D. E. Fan C. Y. Ren H. Y. Cyr D. M. (2011). The endoplasmic reticulum-associated Hsp40 DNAJB12 and Hsc70 cooperate to facilitate RMA1 E3-dependent degradation of nascent CFTRDeltaF508. *Mol. Biol. Cell* 22 301–314. 10.1091/mbc.E10-09-0760 21148293 PMC3031462

[B35] Haardt M. Benharouga M. Lechardeur D. Kartner N. Lukacs G. L. (1999). C-terminal truncations destabilize the cystic fibrosis transmembrane conductance regulator without impairing its biogenesis. A novel class of mutation. *J. Biol. Chem.* 274 21873–21877. 10.1074/jbc.274.31.21873 10419506

[B36] Harris W. T. Muhlebach M. S. Oster R. A. Knowles M. R. Clancy J. P. Noah T. L. (2011). Plasma TGF-β^1^ in pediatric cystic fibrosis: potential biomarker of lung disease and response to therapy. *Pediatr. Pulmonol.* 46 688–695. 10.1002/ppul.21430 21337732 PMC3115503

[B37] Hegde R. N. Parashuraman S. Iorio F. Ciciriello F. Capuani F. Carissimo A. (2015). Unravelling druggable signalling networks that control F508del-CFTR proteostasis. *Elife* 4:e10365. 10.7554/eLife.10365 26701908 PMC4749566

[B38] Hou X. Wei H. Rajagopalan C. Jiang H. Wu Q. Zaman K. (2018). Dissection of the Role of VIMP in endoplasmic reticulum-associated degradation of CFTRΔF508. *Sci. Rep.* 8:4764. 10.1038/s41598-018-23284-8 29555962 PMC5859151

[B39] Hutt D. M. Herman D. Rodrigues A. P. Noel S. Pilewski J. M. Matteson J. (2010). Reduced histone deacetylase 7 activity restores function to misfolded CFTR in cystic fibrosis. *Nat. Chem. Biol.* 6 25–33. 10.1038/nchembio.275 19966789 PMC2901172

[B40] Kim S. J. Skach W. R. (2012). Mechanisms of CFTR folding at the endoplasmic reticulum. *Front. Pharmacol.* 3:201 10.3389/fphar.2012.00201 PMC3521238 23248597

[B41] Kimura T. Kawabe H. Jiang C. Zhang W. Xiang Y. Y. Lu C. (2011). Deletion of the ubiquitin ligase Nedd4L in lung epithelia causes cystic fibrosis-like disease. *Proc. Natl. Acad. Sci. U.S.A.* 108 3216–3221. 10.1073/pnas.1010334108 21300902 PMC3044364

[B42] Kleizen B. van Vlijmen T. de Jonge H. R. Braakman I. (2005). Folding of CFTR is predominantly cotranslational. *Mol. Cell.* 20 277–287. 10.1016/j.molcel.2005.09.007 16246729

[B43] Koch C. (2002). Early infection and progression of cystic fibrosis lung disease. *Pediatr. Pulmonol.* 34 232–236. 10.1002/ppul.10135 12203855

[B44] Koeppen K. Chapline C. Sato J. D. Stanton B. A. (2012). Nedd4-2 does not regulate wt-CFTR in human airway epithelial cells. *Am. J. Physiol. Lung. Cell Mol. Physiol.* 303 L720–L727. 10.1152/ajplung.00409.2011 22904170 PMC3469630

[B45] Kongsuphol P. Cassidy D. Hieke B. Treharne K. J. Schreiber R. Mehta A. (2009). Mechanistic insight into control of CFTR by AMPK. *J. Biol. Chem.* 284 5645–5653. 10.1074/jbc.M806780200 19095655 PMC2645823

[B46] Lobo M. J. Amaral M. D. Zaccolo M. Farinha C. M. (2016). EPAC1 activation by cAMP stabilizes CFTR at the membrane by promoting its interaction with NHERF1. *J. Cell Sci.* 129 2599–2612. 10.1242/jcs.185629 27206858

[B47] Loo M. A. Jensen T. J. Cui L. Hou Y. Chang X. B. Riordan J. R. (1998). Perturbation of Hsp90 interaction with nascent CFTR prevents its maturation and accelerates its degradation by the proteasome. *EMBO J.* 17 6879–6887. 10.1093/emboj/17.23.6879 9843494 PMC1171036

[B48] Lopes-Pacheco M. (2016). CFTR modulators: shedding light on precision medicine for cystic fibrosis. *Front. Pharmacol.* 7:275. 10.3389/fphar.2016.00275 27656143 PMC5011145

[B49] Loureiro C. A. Matos A. M. Dias-Alves Â Pereira J. F. Uliyakina I. Barros P. (2015). A molecular switch in the scaffold NHERF1 enables misfolded CFTR to evade the peripheral quality control checkpoint. *Sci. Signal* 8:ra48. 10.1126/scisignal.aaa1580 25990958

[B50] Luciani A. Villella V. R. Esposito S. Gavina M. Russo I. Silano M. (2012). Targeting autophagy as a novel strategy for facilitating the therapeutic action of potentiators on ΔF508 cystic fibrosis transmembrane conductance regulator. *Autophagy* 8 1657–1672. 10.4161/auto.21483 22874563 PMC3494594

[B51] Lukacs G. L. Chang X. B. Bear C. Kartner N. Mohamed A. Riordan J. R. (1993). The delta F508 mutation decreases the stability of cystic fibrosis transmembrane conductance regulator in the plasma membrane. Determination of functional half-lives on transfected cells. *J. Biol. Chem.* 268 21592–21598. 7691813

[B52] Luz S. Cihil K. M. Brautigan D. L. Amaral M. D. Farinha C. M. Swiatecka-Urban A. (2014). LMTK2-mediated phosphorylation regulates CFTR endocytosis in human airway epithelial cells. *J. Biol. Chem.* 289 15080–15093. 10.1074/jbc.M114.563742 24727471 PMC4031558

[B53] Luz S. Kongsuphol P. Mendes A. I. Romeiras F. Sousa M. Schreiber R. (2011). Contribution of casein kinase 2 and spleen tyrosine kinase to CFTR trafficking and protein kinase A-induced activity. *Mol. Cell. Biol.* 31 4392–4404. 10.1128/MCB.05517-11 21930781 PMC3209257

[B54] Marozkina N. V. Yemen S. Borowitz M. Liu L. Plapp M. Sun F. (2010). Hsp 70/Hsp 90 organizing protein as a nitrosylation target in cystic fibrosis therapy. *Proc. Natl. Acad. Sci. U.S.A.* 107 11393–11398. 10.1073/pnas.0909128107 20534503 PMC2895117

[B55] Matsumura Y. Sakai J. Skach W. R. (2013). Endoplasmic reticulum protein quality control is determined by cooperative interactions between Hsp/c70 protein and the CHIP E3 ligase. *J. Biol. Chem.* 288 31069–31079. 10.1074/jbc.M113.479345 23990462 PMC3829420

[B56] Mazumdar M. Christiani D. C. Biswas S. K. Ibne-Hasan O. S. Kapur K. Hug C. (2015). Elevated sweat chloride levels due to arsenic toxicity. *N. Engl. J. Med.* 372 582–584. 10.1056/NEJMc1413312 25651269 PMC4368195

[B57] McClure M. L. Barnes S. Brodsky J. L. Sorscher E. J. (2016). Trafficking and function of the cystic fibrosis transmembrane conductance regulator: a complex network of posttranslational modifications. *Am. J. Physiol. Lung. Cell Mol. Physiol.* 311 L719–L733. 10.1152/ajplung.00431.2015 27474090 PMC5142128

[B58] Meacham G. C. Lu Z. King S. Sorscher E. Tousson A. Cyr D. M. (1999). The Hdj-2/Hsc70 chaperone pair facilitates early steps in CFTR biogenesis. *EMBO J.* 18 1492–1505. 10.1093/emboj/18.6.1492 10075921 PMC1171238

[B59] Meacham G. C. Patterson C. Zhang W. Younger J. M. Cyr D. M. (2001). The Hsc70 co-chaperone CHIP targets immature CFTR for proteasomal degradation. *Nat. Cell Biol.* 3 100–105. 10.1038/35050509 11146634

[B60] Mendes A. I. Matos P. Moniz S. Luz S. Amaral M. D. Farinha C. M. (2011). Antagonistic regulation of cystic fibrosis transmembrane conductance regulator cell surface expression by protein kinases WNK4 and spleen tyrosine kinase. *Mol. Cell. Biol.* 31 4076–4086. 10.1128/MCB.05152-11 21807898 PMC3187369

[B61] Morito D. Hirao K. Oda Y. Hosokawa N. Tokunaga F. Cyr D. M. (2008). Gp78 cooperates with RMA1 in endoplasmic reticulum-associated degradation of CFTRDeltaF508. *Mol. Biol. Cell* 19 1328–1336. 10.1091/mbc.E07-06-0601 18216283 PMC2291415

[B62] Nery F. C. Armata I. A. Farley J. E. Cho J. A. Yaqub U. Chen P. (2011). TorsinA participates in endoplasmic reticulum-associated degradation. *Nat. Commun.* 2:393. 10.1038/ncomms1383 21750546 PMC3529909

[B63] Odunuga O. O. Longshaw V. M. Blatch G. L. (2004). Hop: more than an Hsp70/Hsp90 adaptor protein. *Bioessays* 26 1058–1068. 10.1002/bies.20107 15382137

[B64] Okiyoneda T. Barrière H. Bagdány M. Rabeh W. M. Du K. Höhfeld J. (2010). Peripheral protein quality control removes unfolded CFTR from the plasma membrane. *Science* 329 805–810. 10.1126/science.1191542 20595578 PMC5026491

[B65] Okiyoneda T. Harada K. Takeya M. Yamahira K. Wada I. Shuto T. (2004). Delta F508 CFTR pool in the endoplasmic reticulum is increased by calnexin overexpression. *Mol. Biol. Cell* 15 563–574. 10.1091/mbc.e03-06-0379 14595111 PMC329241

[B66] Okiyoneda T. Niibori A. Harada K. Kohno T. Michalak M. Duszyk M. (2008). Role of calnexin in the ER quality control and productive folding of CFTR; differential effect of calnexin knockout on wild-type and DeltaF508 CFTR. *Biochim. Biophys. Acta* 1783 1585–1594. 10.1016/j.bbamcr.2008.04.002 18457676

[B67] Okiyoneda T. Veit G. Dekkers J. F. Bagdany M. Soya N. Xu H. (2013). Mechanism-based corrector combination restores ΔF508-CFTR folding and function. *Nat. Chem. Biol.* 9 444–454. 10.1038/nchembio.1253 23666117 PMC3840170

[B68] Okiyoneda T. Veit G. Sakai R. Aki M. Fujihara T. Higashi M. (2018). Chaperone-independent peripheral quality control of CFTR by RFFL E3 ligase. *Dev. Cell* 44 694–708.e7. 10.1016/j.devcel.2018.02.001 29503157 PMC6447300

[B69] Phuan P. W. Veit G. Tan J. A. Finkbeiner W. E. Lukacs G. L. Verkman A. S. (2015). Potentiators of defective ΔF508-CFTR gating that do not interfere with corrector action. *Mol. Pharmacol.* 88 791–799. 10.1124/mol.115.099689 26245207 PMC4576684

[B70] Pind S. Riordan J. R. Williams D. B. (1994). Participation of the endoplasmic reticulum chaperone calnexin (p88, IP90) in the biogenesis of the cystic fibrosis transmembrane conductance regulator. *J. Biol. Chem.* 269 12784–12788. 7513695

[B71] Qian Z. Xu X. Amacher J. F. Madden D. R. Cormet-Boyaka E. Pei D. (2015). Intracellular delivery of peptidyl ligands by reversible cyclization: discovery of a PDZ domain inhibitor that rescues CFTR activity. *Angew. Chem. Int. Ed. Engl.* 54 5874–5878. 10.1002/anie.201411594 25785567 PMC4424104

[B72] Rabeh W. M. Bossard F. Xu H. Okiyoneda T. Bagdany M. Mulvihill C. M. (2012). Correction of both NBD1 energetics and domain interface is required to restore ΔF508 CFTR folding and function. *Cell* 148 150–163. 10.1016/j.cell.2011.11.024 22265408 PMC3431169

[B73] Ramachandran S. Osterhaus S. R. Parekh K. R. Jacobi A. M. Behlke M. A. McCray P. B. (2016). SYVN1, NEDD8, and FBXO2 proteins regulate ΔF508 cystic fibrosis transmembrane conductance regulator (CFTR). Ubiquitin-mediated proteasomal degradation. *J. Biol. Chem.* 291 25489–25504. 10.1074/jbc.M116.754283 27756846 PMC5207249

[B74] Rasmussen J. E. Sheridan J. T. Polk W. Davies C. M. Tarran R. (2014). Cigarette smoke-induced Ca2 + release leads to cystic fibrosis transmembrane conductance regulator (CFTR) dysfunction. *J. Biol. Chem.* 289 7671–7681. 10.1074/jbc.M113.545137 24448802 PMC3953278

[B75] Ren H. Y. Grove D. E. De La Rosa O. Houck S. A. Sopha P. Van Goor F. (2013). VX-809 corrects folding defects in cystic fibrosis transmembrane conductance regulator protein through action on membrane-spanning domain 1. *Mol. Biol. Cell* 24 3016–3024. 10.1091/mbc.E13-05-0240 23924900 PMC3784376

[B76] Rennolds J. Butler S. Maloney K. Boyaka P. N. Davis I. C. Knoell D. L. (2010). Cadmium regulates the expression of the CFTR chloride channel in human airway epithelial cells. *Toxicol. Sci.* 116 349–358. 10.1093/toxsci/kfq101 20363832 PMC2886859

[B77] Rosser M. F. Grove D. E. Chen L. Cyr D. M. (2008). Assembly and misassembly of cystic fibrosis transmembrane conductance regulator: folding defects caused by deletion of F508 occur before and after the calnexin-dependent association of membrane spanning domain (MSD). 1 and MSD2. *Mol. Biol. Cell* 19 4570–4579. 10.1091/mbc.E08-04-0357 18716059 PMC2575159

[B78] Rotin D. Staub O. (2012). Nedd4-2 and the regulation of epithelial sodium transport. *Front. Physiol.* 3:212 10.3389/fphys.2012.00212 PMC3380336 22737130

[B79] Sharma M. Pampinella F. Nemes C. Benharouga M. So J. Du K. (2004). Misfolding diverts CFTR from recycling to degradation: quality control at early endosomes. *J. Cell Biol.* 164 923–933. 10.1083/jcb.200312018 15007060 PMC2172283

[B80] Silvis M. R. Picciano J. A. Bertrand C. Weixel K. Bridges R. J. Bradbury N. A. (2003). A mutation in the cystic fibrosis transmembrane conductance regulator generates a novel internalization sequence and enhances endocytic rates. *J. Biol. Chem.* 278 11554–11560. 10.1074/jbc.M212843200 12529365

[B81] Snodgrass S. M. Cihil K. M. Cornuet P. K. Myerburg M. M. Swiatecka-Urban A. (2013). Tgf-β1 inhibits Cftr biogenesis and prevents functional rescue of ΔF508-Cftr in primary differentiated human bronchial epithelial cells. *PLoS One* 8:e63167. 10.1371/journal.pone.0063167 23671668 PMC3650079

[B82] Sondo E. Falchi F. Caci E. Ferrera L. Giacomini E. Pesce E. (2018). Pharmacological inhibition of the ubiquitin ligase RNF5 rescues F508del-CFTR in cystic fibrosis airway epithelia. *Cell Chem. Biol.* 25 891–905.e8. 10.1016/j.chembiol.2018.04.010 29754957

[B83] Sun H. Harris W. T. Kortyka S. Kotha K. Ostmann A. J. Rezayat A. (2014). Tgf-beta downregulation of distinct chloride channels in cystic fibrosis-affected epithelia. *PLoS One* 9:e106842. 10.1371/journal.pone.0106842 25268501 PMC4182049

[B84] Swiatecka-Urban A. Brown A. Moreau-Marquis S. Renuka J. Coutermarsh B. Barnaby R. (2005). The short apical membrane half-life of rescued {Delta}F508-cystic fibrosis transmembrane conductance regulator (CFTR).results from accelerated endocytosis of {Delta}F508-CFTR in polarized human airway epithelial cells. *J. Biol. Chem.* 280 36762–36772. 10.1074/jbc.M508944200 16131493

[B85] Swiatecka-Urban A. Moreau-Marquis S. Maceachran D. P. Connolly J. P. Stanton C. R. Su J. R. (2006). *Pseudomonas aeruginosa* inhibits endocytic recycling of CFTR in polarized human airway epithelial cells. *Am. J. Physiol. Cell Physiol.* 290 C862–C872. 10.1152/ajpcell.00108.2005 16236828

[B86] Thelin W. R. Chen Y. Gentzsch M. Kreda S. M. Sallee J. L. Scarlett C. O. (2007). Direct interaction with filamins modulates the stability and plasma membrane expression of CFTR. *J. Clin. Invest.* 117 364–374. 10.1172/JCI30376 17235394 PMC1765518

[B87] Trzcinska-Daneluti A. M. Nguyen L. Jiang C. Fladd C. Uehling D. Prakesch M. (2012). Use of kinase inhibitors to correct ΔF508-CFTR function. *Mol. Cell. Proteomics* 11 745–757. 10.1074/mcp.M111.016626 22700489 PMC3434788

[B88] Turnbull E. L. Rosser M. F. Cyr D. M. (2007). The role of the UPS in cystic fibrosis. *BMC Biochem.* 8(Suppl. 1):S11. 10.1186/1471-2091-8-S1-S11 18047735 PMC2106362

[B89] Van Goor F. Hadida S. Grootenhuis P. D. Burton B. Stack J. H. Straley K. S. (2011). Correction of the F508del-CFTR protein processing defect in vitro by the investigational drug VX-809. *Proc. Natl. Acad. Sci. U.S.A.* 108 18843–18848. 10.1073/pnas.1105787108 21976485 PMC3219147

[B90] Van Goor F. Yu H. Burton B. Hoffman B. J. (2014). Effect of ivacaftor on CFTR forms with missense mutations associated with defects in protein processing or function. *J. Cyst. Fibros* 13 29–36. 10.1016/j.jcf.2013.06.008 23891399

[B91] Varga K. Goldstein R. F. Jurkuvenaite A. Chen L. Matalon S. Sorscher E. J. (2008). Enhanced cell-surface stability of rescued DeltaF508 cystic fibrosis transmembrane conductance regulator (CFTR) by pharmacological chaperones. *Biochem. J.* 410 555–564. 10.1042/BJ20071420 18052931 PMC3939615

[B92] Veit G. Avramescu R. G. Chiang A. N. Houck S. A. Cai Z. Peters K. W. (2016a). From CFTR biology toward combinatorial pharmacotherapy: expanded classification of cystic fibrosis mutations. *Mol. Biol. Cell* 27 424–433. 10.1091/mbc.E14-04-0935 26823392 PMC4751594

[B93] Veit G. Avramescu R. G. Perdomo D. Phuan P. W. Bagdany M. Apaja P. M. (2014). Some gating potentiators, including VX-770, diminish ΔF508-CFTR functional expression. *Sci. Transl. Med.* 6:246ra97. 10.1126/scitranslmed.3008889 25101887 PMC4467693

[B94] Veit G. Oliver K. Apaja P. M. Perdomo D. Bidaud-Meynard A. Lin S. T. (2016b). Ribosomal stalk protein silencing partially corrects the ΔF508-CFTR functional expression defect. *PLoS Biol.* 14:e1002462. 10.1371/journal.pbio.1002462 27168400 PMC4864299

[B95] Venerando A. Franchin C. Cant N. Cozza G. Pagano M. A. Tosoni K. (2013). Detection of phospho-sites generated by protein kinase CK2 in CFTR: mechanistic aspects of Thr1471 phosphorylation. *PLoS One* 8:e74232. 10.1371/journal.pone.0074232 24058532 PMC3776838

[B96] Villella V. R. Esposito S. Bruscia E. M. Maiuri M. C. Raia V. Kroemer G. (2013). Targeting the intracellular environment in cystic fibrosis: restoring autophagy as a novel strategy to circumvent the CFTR defect. *Front. Pharmacol.* 4:1. 10.3389/fphar.2013.00001 23346057 PMC3549520

[B97] Wang H. Brautigan D. L. (2006). Peptide microarray analysis of substrate specificity of the transmembrane Ser/Thr kinase KPI-2 reveals reactivity with cystic fibrosis transmembrane conductance regulator and phosphorylase. *Mol. Cell. Proteomics* 5 2124–2130. 10.1074/mcp.M600188-MCP200 16887929

[B98] Wang Y. Liu J. Loizidou A. Bugeja L. A. Warner R. Hawley B. R. (2014). CFTR potentiators partially restore channel function to A561E-CFTR, a cystic fibrosis mutant with a similar mechanism of dysfunction as F508del-CFTR. *Br. J. Pharmacol.* 171 4490–4503. 10.1111/bph.12791 24902474 PMC4209154

[B99] Wang Y. Loo T. W. Bartlett M. C. Clarke D. M. (2007). Correctors promote maturation of cystic fibrosis transmembrane conductance regulator (CFTR)-processing mutants by binding to the protein. *J. Biol. Chem.* 282 33247–33251. 10.1074/jbc.C700175200 17911111

[B100] Wolde M. Fellows A. Cheng J. Kivenson A. Coutermarsh B. Talebian L. (2007). Targeting CAL as a negative regulator of DeltaF508-CFTR cell-surface expression: an RNA interference and structure-based mutagenetic approach. *J. Biol. Chem.* 282 8099–8109. 10.1074/jbc.M611049200 17158866

[B101] Yang Y. Janich S. Cohn J. A. Wilson J. M. (1993). The common variant of cystic fibrosis transmembrane conductance regulator is recognized by hsp70 and degraded in a pre-Golgi nonlysosomal compartment. *Proc. Natl. Acad. Sci. U.S.A.* 90 9480–9484. 10.1073/pnas.90.20.9480 7692448 PMC47592

[B102] Ye S. Cihil K. Stolz D. B. Pilewski J. M. Stanton B. A. Swiatecka-Urban A. (2010). c-Cbl facilitates endocytosis and lysosomal degradation of cystic fibrosis transmembrane conductance regulator in human airway epithelial cells. *J. Biol. Chem.* 285 27008–27018. 10.1074/jbc.M110.139881 20525683 PMC2930700

[B103] Younger J. M. Chen L. Ren H. Y. Rosser M. F. Turnbull E. L. Fan C. Y. (2006). Sequential quality-control checkpoints triage misfolded cystic fibrosis transmembrane conductance regulator. *Cell* 126 571–582. 10.1016/j.cell.2006.06.041 16901789

[B104] Zaman K. Bennett D. Fraser-Butler M. Greenberg Z. Getsy P. Sattar A. (2014). S-Nitrosothiols increases cystic fibrosis transmembrane regulator expression and maturation in the cell surface. *Biochem. Biophys. Res. Commun.* 443 1257–1262. 10.1016/j.bbrc.2013.12.130 24393850 PMC3974270

